# The Role of O-GlcNAcylation in Perivascular Adipose Tissue Dysfunction of Offspring of High-Fat Diet-Fed Rats

**DOI:** 10.1159/000458422

**Published:** 2017-04-04

**Authors:** Karolina E. Zaborska, Gillian Edwards, Clare Austin, Mark Wareing

**Affiliations:** ^a^Institute of Cardiovascular Sciences, University of Manchester, Manchester, UK; ^b^Maternal and Fetal Health Research Centre, Institute of Human Development, University of Manchester, Manchester, UK; ^c^Faculty of Health and Social Care, Edge Hill University, Ormskirk, UK

**Keywords:** Perivascular adipose tissue, O-GlcNAcylation, Prenatal programming, AMP-activated protein kinase

## Abstract

Perivascular adipose tissue (PVAT), which reduces vascular contractility, is dysfunctional in the male offspring of rats fed a high-fat diet (HFD), partially due to a reduced NO bioavailability. O-GlcNAcylation of eNOS decreases its activity, thus we investigated the role of O-GlcNAcylation in the prenatal programming of PVAT dysfunction. Female Sprague-Dawley rats were fed either a control (10% fat) or an obesogenic HFD (45% fat) diet for 12 weeks prior to mating, and throughout pregnancy and lactation. Offspring were weaned onto the control diet and were killed at 12 and 24 weeks of age. Mesenteric arteries from the 12-week-old offspring of HFD dams (HFDO) contracted less to U46619; these effects were mimicked by glucosamine in control arteries. PVAT from 12- and 24-week-old controls, but not from HFDO, exerted an anticontractile effect. Glucosamine attenuated the anticontractile effect of PVAT in the vessels from controls but not from HFDO. AMP-activated protein kinase (AMPK) activation (with A769662) partially restored an anticontractile effect in glucosamine-treated controls and HFDO PVAT. Glucosamine decreased AMPK activity and expression in HFDO PVAT, although phosphorylated eNOS expression was only reduced in that from males. The loss of anticontractile effect of HFDO PVAT is likely to result from increased O-GlcNAcylation, which decreased AMPK activity and, in males, decreased NO bioavailability.

## Introduction

Obesity levels have been rising over the past 2 decades [1]. The numbers of overweight/obese children have also increased [2] and this has significant health care ramifications since overweight children have an increased risk of being obese as adults [2, 3, 4]. Maternal obesity can influence the health of offspring, the so-called “foetal programming of disease” [5, 6, 7]. Offspring of obese mothers and dams can be programmed in utero to develop obesity and cardiovascular dysfunction later in life [8, 9, 10, 11]. However, the mechanism(s) by which maternal obesity programs offspring to develop cardiometabolic disorders is still unknown.

Vascular contractility is modulated by the endothelium through balancing the release of vasodilator and vasoconstrictor agents. Endothelial dysfunction is associated with obesity [12, 13] and is clearly linked to decreased nitric oxide (NO) bioavailability [7, 14]. Interestingly, endothelial dysfunction also occurs in the offspring of dams fed an obesogenic diet during their pregnancy [15].

Obesity-linked hypertension is proposed to be associated with loss of perivascular adipose tissue (PVAT) function [16]. Most blood vessels are surrounded by PVAT [17], an endocrine organ which secrets numerous bioactive proteins (adipokines) [18] that modulate vascular function. The predominantly anticontractile effect of PVAT in healthy subjects results from the release of PVAT-derived relaxant factors, including NO [19], which more than offset the effects of PVAT-derived contractile factors. Obesity is associated with a diminished PVAT anticontractile effect and decreased endothelium-dependent vasodilation [20, 21, 22]. Impaired adipokine production, inflammation, and oxidative stress have been proposed to be responsible for the reduced anticontractile effect of PVAT observed in obesity [21, 22]. Our group has previously demonstrated that this anticontractile effect of PVAT is lost in the male offspring of rats that have been fed a high-fat diet (HFD dams) [23].

Protein O-GlcNAcylation is a dynamic, reversible process that attaches the O-linked β-*N*-acetylglucosamine (O-GlcNAc) to serine/threonine residues of proteins. Under normal conditions, 2–5% of glucose that is taken up by cells enters the hexosamine biosynthetic pathway in which glucose is utilised by glutamine fructose-6-phosphate amidotransferase to uridine diphosphate *N*-acetylglucosamine. Glycosyltransferase then mediates the attachment of the O-GlcNAc to serine/threonine residues of target proteins. Hyperglycaemia and hyperinsulinemia increase protein O-GlcNAcylation, which has been suggested to mediate the adverse effects of insulin resistance and diabetes on vascular function [24]. We have previously found that the plasma insulin levels are elevated in the male offspring of obese dams [23], which would suggest that protein O-GlcNAcylation might be increased in these animals. O-GlcNAcylation of eNOS is known to decrease its activity [25]and leads to impaired NO-mediated arterial dilation [26], and we have previously shown that the anticontractile effect of PVATis lost in male offspring of HFD dams due to reduced NO bioavailability [23].

AMP-activated protein kinase (AMPK) is an energy sensor involved in the regulation of glucose, lipid, and protein metabolism [27], and in obesity dysregulation of these processes is associated with reduced AMPK activity [28]. In the vasculature, AMPK phosphorylates and activates eNOS [29]. In animal models of the metabolic syndrome, AMPK activation reverses insulin resistance [30, 31, 32] and partially reverses the associated endothelial dysfunction by increasing the phosphorylation of eNOS [33]. AMPK activation also reduces vascular contractions in PVAT-intact arteries from the offspring of control and HFD dams [23], so changes in its activity could contribute to the effect of HFD programming of PVAT function in offspring.

We hypothesised that prenatal programming of PVAT would be associated with increased O-GlcNAcylation of eNOS and AMPK. Thus, the aim of this study was to identify changes in the modulation of vascular contraction by PVAT in the offspring of HFD-fed rats and determine whether O-GlcNAcylation of eNOS and AMPK play a role in these changes. In order to avoid the modification of vascular tone by O-GlcNAcylation of myocyte proteins, PVAT was dissected free from artery segments prior to pharmacological modulation, then re-introduced into the myograph bath before the construction of concentration-response relationships.

## Methods

### Animals

All animal procedures complied with the United Kingdom Animals (Scientific Procedures) Act. Animals were housed under a 12-h light/12-h dark cycle and provided with food and water ad libitum. Female Sprague-Dawley rats (130–165 g, Charles River, Harlow, UK) were fed an obesogenic 45% fat diet (HFD; 824018, SDS Diets, Witham, UK) for 12 weeks or a control 10% fat diet (824050, SDS diets) for the 12-week period prior to mating, and continued on their respective diets during the pregnancy and the 4-week lactation period. To reduce variability due to the availability of breast milk, litter sizes were reduced to 4 males and 4 females per dam at postnatal day 6. At weaning, the offspring were fed the control 10% fat diet until sacrifice at either 12 or 24 weeks of age. Body weight was recorded fortnightly in the dams and weekly in the offspring.

### Blood Pressure

Systolic and diastolic blood pressures were measured in conscious rats using a CODA tail-cuff blood pressure monitoring system (Kent Scientific, Torrington, CT, USA). All animals were acclimatized to the monitoring procedure on 3 occasions prior to blood pressure measurements. Blood pressure was recorded in all animals between 8:00 and 11:00 a.m. to avoid diurnal variation.

### Blood Glucose Measurement

Fasting blood samples were taken from conscious rats by lateral tail vein sampling for the assessment of blood glucose concentration (automatic blood glucose monitor, Contour, Bayer Consumer Care AG, Basel, Switzerland) and plasma insulin levels (Rat Insulin ELISA, Alpco, Salem, NH, USA).

### Wire Myography

Overnight-fasted animals were killed by CO_2_ inhalation followed by mechanical disruption of the diaphragm. Female offspring were sacrificed during the oestrous stage of their cycle. The weight of epididymal and inguinal fat pads was recorded. The mesenteric vascular bed was isolated and immediately placed in ice-cold physiological salt solution (PSS; 119 mmol/L NaCl, 4.7 mmol/L KCl, 1.17 mmol/L MgSO_4_, 25 mmol/L NaHCO_3_, 1.17 mmol/L KH_2_PO_4_, 0.03 mmol/L K2 EDTA, 5.5 mmol/L glucose and 1.6 mmol/L CaCl_2_). Second-order mesenteric arteries, cleaned of all fat and connective tissue (-PVAT), were mounted on 40-µm wires in a small vessel wire myograph with or without the exogenous PVAT (ePVAT) suspended in close proximity to the vessel. Vessels were then equilibrated in PSS at 37°C (gassed with 5% CO_2_ in air) for 30 min then tensioned to the equivalent of an internal pressure of 80 mm Hg, as previously described [34]. Changes in vessel tension were recorded continuously using Chart 5 Pro Software (ADInstruments, Oxford, UK). Vessel viability was assessed using high-potassium (60 mmol/L) PSS (KPSS).

### Pharmacological Modulation

Arteries or PVAT (in separate baths) were incubated with an O-GlcNAcylator, glucosamine (10 mmol/L; Sigma-Aldrich, Gillingham, UK), for 2 h with or without an AMPK activator, A769662 (6,7-dihydro-4-hydroxy-3-(2′-hydroxy[1,1′-biphenyl]-4-yl)-6-oxo-thieno[2, 3-b]pyridine-5-carbonitrile; 10 µmol/L; R&D Systems, Abingdon, UK), for 1 h. ePVAT was placed on top of the vessels before cumulative concentration-response curves were constructed to a thromboxane A_2_ agonist U46619 (9,11-dideoxy-9a,11a-methanoepoxy prostaglandin F_2a_; 10 nmol/L to 3 µmol/L; R&D Systems). Separation of the PVAT prior to drug exposure and its subsequent re-introduction into the myograph bath allowed an investigation of the effects of pharmacological modulation of the PVAT without directly influencing vascular tone. Arteries precontracted with U46619 were relaxed with acetylcholine (Sigma-Aldrich) to assess functional endothelial integrity.

To assess the effect of glucosamine on protein expression, 300 mg of PVAT was incubated with 10 mmol/L of glucosamine or vehicle (PSS) at 37°C for 2 h. Pharmacologically modulated PVAT was snap-frozen in liquid nitrogen and stored at —80°C.

### Tissue Lysates

Tissues were lysed by homogenising in RIPA buffer (Sigma-Aldrich) supplemented with protein protease (Roche, Mannheim, Germany) and phosphatase inhibitors (Roche), and the protein content was quantified as previously described [23].

### Western Blot

Proteins (40 µg) were separated on a 7.5% stain-free SDS-polyacrylamide gel by electrophoresis then transferred to mini low fluorescence PVDF membranes (Bio-Rad Laboratories, Hemel Hempstead, UK) and visualised using the Chemidoc MP imaging system (Bio-Rad Laboratories). The membrane was blocked with 3.5% (w/v) TBS-Tween (10 mmol/L Tris pH 8, 150 mmol/L NaCl, 0.1% Tween 2.0) for 1 h at room temperature (RT). Appropriate primary antibody was added and incubated overnight at 4°C (anti-eNOS 1:200, anti-peNOS^ser1177^ 1:200, anti-O-GlcNAc 1:200; Santa Cruz Biotechnology, Dallas, TX, USA; anti-AMPKα 1:1,000 and anti-pAMPKα 1:1,000; Cell Signalling, Hitchin, UK) followed by exposure to a horseradish peroxidase-conjugated secondary antibody for 1 h at RT. Chemiluminescence was performed with Clarity Western ECL Substrate (Bio-Rad Laboratories) and images were obtained using the Chemidoc MP imaging system. Immunoblots were analysed using ImageLab 5.2.1 (Bio-Rad Laboratories) software and adjusted to the total protein of the sample as previously described [35].

### Immunoprecipitation

PVAT was homogenised in immunoprecipitation buffer (50 mmol/L Tris-HCl, pH 7.4 at 4°C; 150 mmol/L NaCl; 50 mmol/L NaF; 5 mmol/L Na_4_P_2_O_7_; 1 mmol/L sodium vanadate; 1 mmol/L EDTA; 1 mmol/L EGTA; 1% glycerol; 1% Triton X-100; 1 mmol/L dithiothreitol; 0.1 mmol/L benzamidine; 0.1 mmol/L phenylmethylsulfonyl fluoride; 5 mg/L soya bean trypsin inhibitor) in a Dounce homogeniser. AMPK was immunoprecipitated from PVAT (45 µg) using AMPKα1 and AMPKα2 antibodies (kindly provided by Dr. Ian Salt) bound to protein G sepharose beads (GE Healthcare, Little Chalfont, UK).

### AMPK Activity Assay

AMPK was immunoprecipitated from PVAT (45 µg) and basal activity was measured using the SAMS substrate peptide as described previously [36]. The results are expressed as nmol ^32^P incorporated into SAMS peptide min^-1^ (mg PVAT lysate)^-1^ normalised to controls.

### Data Analysis

Data are presented as the mean ± SEM. The cumulative concentration-response curves (fitted using a non-linear regression analysis) were expressed as the percentage contraction to 60 mM KPSS.

Statistical analysis was performed using GraphPad Prism v.6 (GraphPad Software, La Jolla, CA, USA) with 2/1-way ANOVA or an unpaired/paired *t* test. *p* < 0.05 values were considered statistically significant.

## Results

### Maternal Characteristics

Prior to pregnancy, the maternal body weight and arterial blood pressure were increased in HFD dams compared to controls, although glucose remained unchanged (online suppl. Fig. 1; for all online suppl. material, see www.karger.com/doi/10.1159/000458422). There was a trend towards increased plasma insulin levels, although this did not reach statistical significance (online suppl. Fig. 1). The litter sizes were similar in control (median 14 pups/dam, range 8–18) and HFD (median 16 pups/dam, range 15–17) dams.

### Offspring Characteristics

**Fig. 1:**
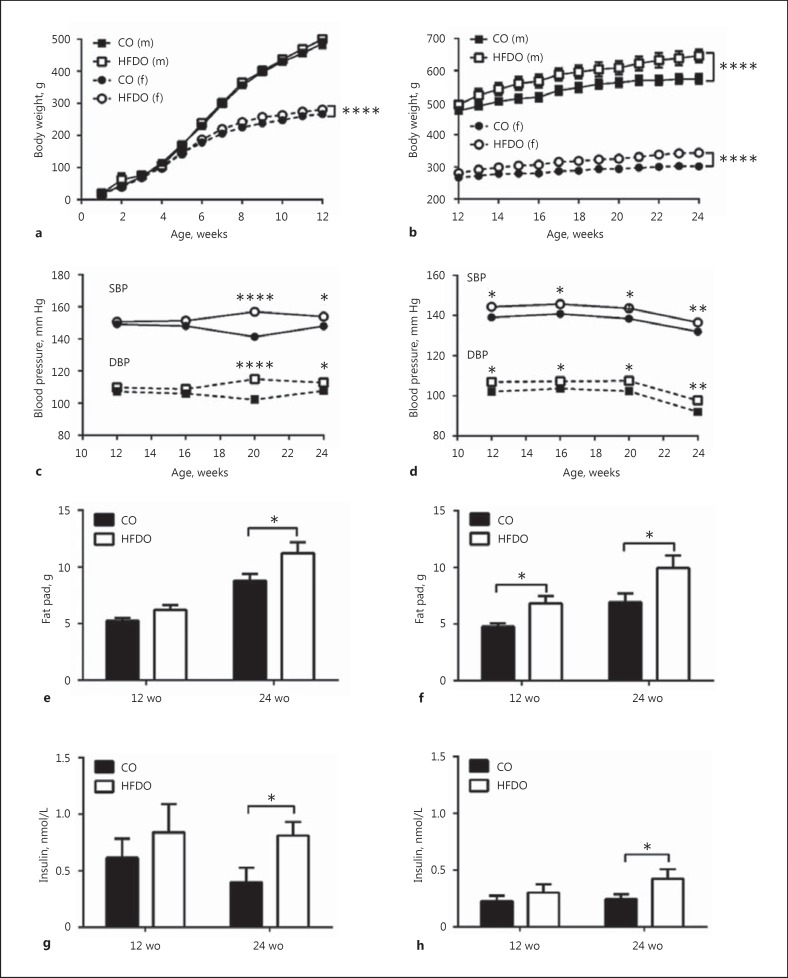
Offspring characteristics. Body weight was increased in 12-week-old female offspring of HFD dams but not in males compared to controls (**a**), whereas at 24 weeks of age the body weight was increased in both male and female offspring of HFD dams (**b**). Arterial blood pressure was increased in the male offspring of HFD dams from week 20 onwards (**c**) but in female offspring it was elevated from week 12 (**d**). Fat pads were larger in 24-week-old male (**e**) and female (**f**) offspring of HFD dams. Plasma insulin levels were increased in both the 24-week-old male (**g**) and female (**h**) offspring of HFD dams. Data are expressed as the mean ± SEM, *n* = 10; * *p* < 0.05, ** *p* < 0.01, **** *p* < 0.0001, 2-way repeated-measures ANOVA or unpaired *t* test, as appropriate. CO, controls; m, males; f, females; wo, weeks old.

**Fig. 2:**
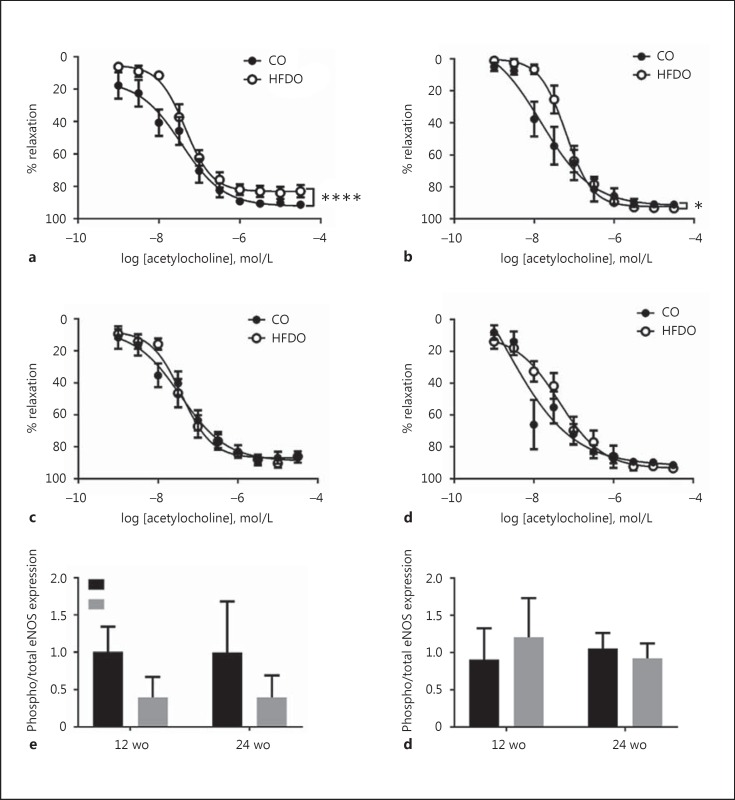
Endothelial function. Vascular relaxation to acetylcholine was reduced in both 12- (**a**) and 24-week-old (**b**) male offspring of HFD dams compared to controls, but not in either 12- (**c**) or 24-week-old (**d**) female offspring. The ratio of phosphorylated to total eNOS expression was reduced in mesenteric arteries from 12- and 24-week-old male offspring of HFD dams compared to controls (**e**), but not in female offspring (**f**). Data are expressed as the mean ± SEM, *n* = 5–10; * *p* < 0.05, **** *p* < 0.0001, 1/2-way ANOVA. CO, controls; wo, weeks old. Black box = CO; Grey box = HFDO.

Body weight was similar between the 12-week-old male offspring of control (12wCO) and HFD (12wHFDO) dams (Fig. 1a), but it was significantly increased in the 24-week-old male offspring of HFD (24wHFDO) dams compared to controls (24wCO; Fig. 1b). Body weight was significantly increased in female HFDO at both 12 (Fig. 1a) and 24 weeks (Fig. 1b) of age. Arterial blood pressure was normal in male HFDO at 12 weeks of age but was elevated from week 20 onwards (Fig. 1c). Systolic and diastolic blood pressures were significantly increased from week 12 onwards in female HFDO (Fig. 1d). Epididymal fat pads weights were increased in male 24wHFDO (Fig. 1e) and inguinal fat pads (Fig. 1f) were larger in female 12wHFDO and 24wHFDO. The plasma insulin levels were significantly elevated in male (Fig. 1g) and female (Fig. 1h) 24wHFDO compared to controls; there was also a trend towards increased plasma insulin concentration in male and female 12wHFDO compared to 12wCO. The endothelial function (assessed by relaxation to acetylcholine) was reduced in male 12 and 24wHFDO compared to controls (Fig. 2a, b), but not in female offspring (Fig. 2c, d). The ratio of phosphorylated to total eNOS expression was reduced in mesenteric arteries from male HFDO compared to controls, but was unchanged in female offspring (Fig. 2e, f). Maternal insulin levels positively correlated with their offspring's insulin levels (online suppl. Fig. 2A, B, C, D).

### Loss of the Anticontractile Effect of PVAT in Offspring of HFD Dams

**Fig. 3:**
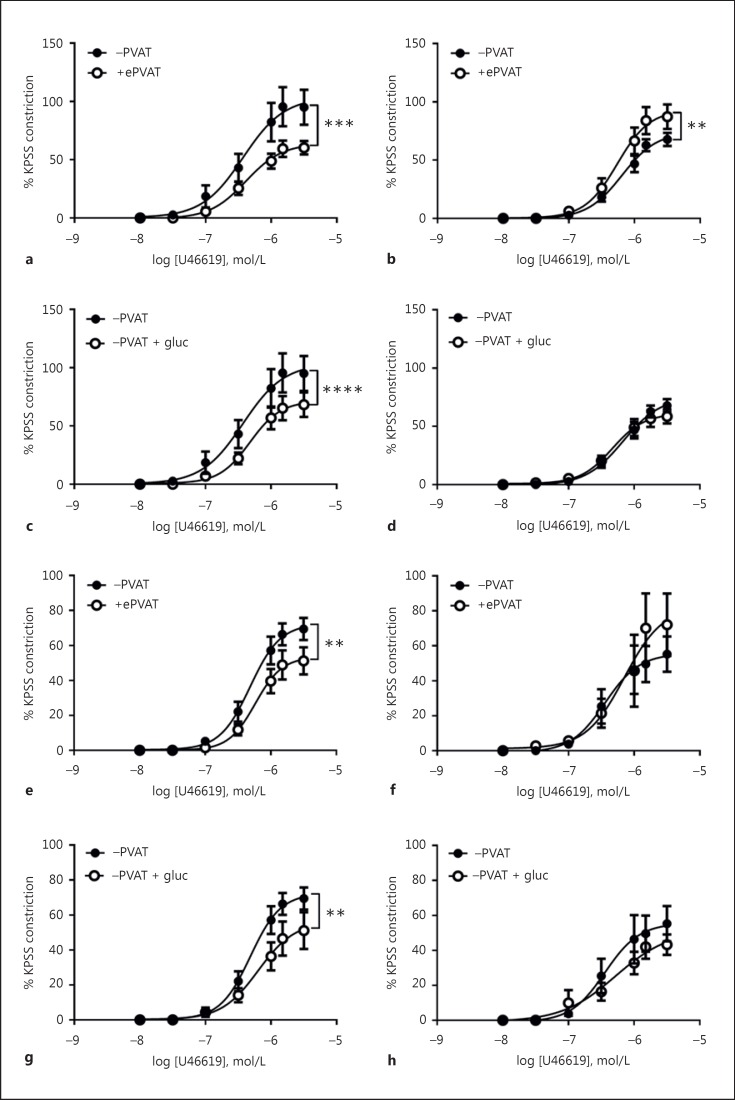
Loss of the anticontractile effect of PVAT in 12-week-old offspring of HFD dams. ePVAT exerted an anticontractile effect on the corresponding PVAT-denuded mesenteric artery segments from 12-week-old male (**a**) and female (**e**) offspring of control but not HFD dams (males, **b**; females, **f**). Pre-incubation of PVAT-denuded male (**c**) and female (**g**) control vessels with 10 mmol/L of glucosamine (gluc) decreased the magnitude of contractions to U46619 to levels similar to those in male (**d**) and female (**h**) HFD vessels. Data are expressed as the mean ± SEM, *n* = 10; ** *p* < 0.01, *** *p* < 0.001, 2-way ANOVA.

PVAT exerted an anticontractile effect in the arteries from male and female 12wCO (Fig. 3a, e, respectively); whereas it was contractile in male HFDO (Fig. 3b) and there was a trend towards PVAT being contractile in female HFDO (Fig. 3f). These changes in PVAT function were also observed in male and female 24wHFDO when compared to their respective controls (online suppl. Fig. 3).

In comparison to responses in age-matched control vessels, in the absence of PVAT, vascular contractions to U46619 were reduced in artery segments from male and female 12wHFDO (*p* < 0.01); a similar reduction was produced in male and female 12wCO vessels by pre-incubation with glucosamine (Fig. 3c, g, respectively). Pre-incubation of male and female 12wHFDO arteries with glucosamine had no effect on vascular contractions (Fig. 3d, h, respectively). However, this reduction in vascular contractions to U46619 were only observed in 12wHFDO but not in 24wHFDO (online suppl. Fig. 3). Emax values for all conditions are summarised in online supplementary Table 1.

**Fig. 4:**
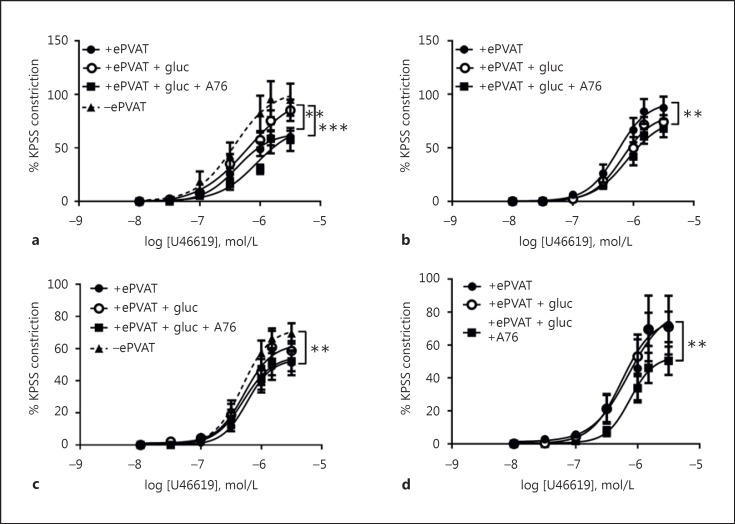
AMPK activation within PVAT partially restored the anticontractile effect of PVAT in the 12-week-old offspring of HFD dams. Pre-incubation of ePVAT with glucosamine (gluc) increased vascular contractions in male control vessels (**a**) but not HFD offspring arteries (**b**); simultaneous AMPK activation (with A769662) within male control (**a**) and HFD (**b**) ePVAT restored its anticontractile properties. Pre-incubation with gluc had no effect on female control ePVAT (**c**) and HFD ePVAT (**d**). However, simultaneous AMPK activation (with A769662) within female HFD (**d**) ePVAT restored its anticontractile properties. Data are expressed as the mean ± SEM, *n* = 10; ** *p* < 0.01, *** *p* < 0.001, 2-way ANOVA.

PVAT from male 12wCO or 24wCO pre-incubated with glucosamine increased vascular contractions of PVAT-denuded vessels, whereas, despite the presence of glucosamine, simultaneous AMPK activation within PVAT restored anticontractile capability (Fig. 4a; online suppl. Fig. 4). PVAT from female 12wCO or 24wCO was no longer anticontractile when pre-incubated with glucosamine (Fig. 4c; online suppl. Fig. 4). PVAT from male and female 12wHFDO pre-incubated with glucosamine had no effect on PVAT-denuded vessels but simultaneous AMPK activation within PVAT reduced vascular contractions to U46619 and partially restored the anticontractile effect of PVAT (Fig. 4b, d, respectively). In contrast, after the pre-incubation of PVAT from female 24wHFDO vessels with glucosamine, the PVAT enhanced vascular contractions to U46619, although if co-incubated with an AMPK activator the PVAT became anticontractile (online suppl. Fig. 4).

The sensitivity to U46619 was reduced in mesenteric arteries with ePVAT pre-incubated with glucosamine and the AMPK activator compared to arteries with ePVAT from female 24wHFDO, but not in any other groups (online suppl. Table 2).

### O-GlcNAcylation in PVAT and Mesenteric Arteries: Effect of Glucosamine

**Fig. 5:**
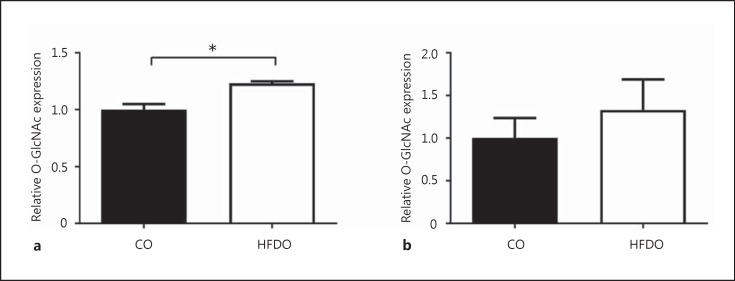
Glucosamine-enhanced O-GlcNAcylation in PVAT-denuded mesenteric arteries. The total O-GlcNAcylation of protein was increased in 12-week-old male (**a**) HFD arteries compared to their respective controls (CO). There was a trend towards increased expression of O-GlcNac in arteries from 12-week-old female HFD offspring (**b**). Data are expressed as the mean ± SEM, *n* = 4; * *p* < 0.05, Student *t* test.

**Fig. 6:**
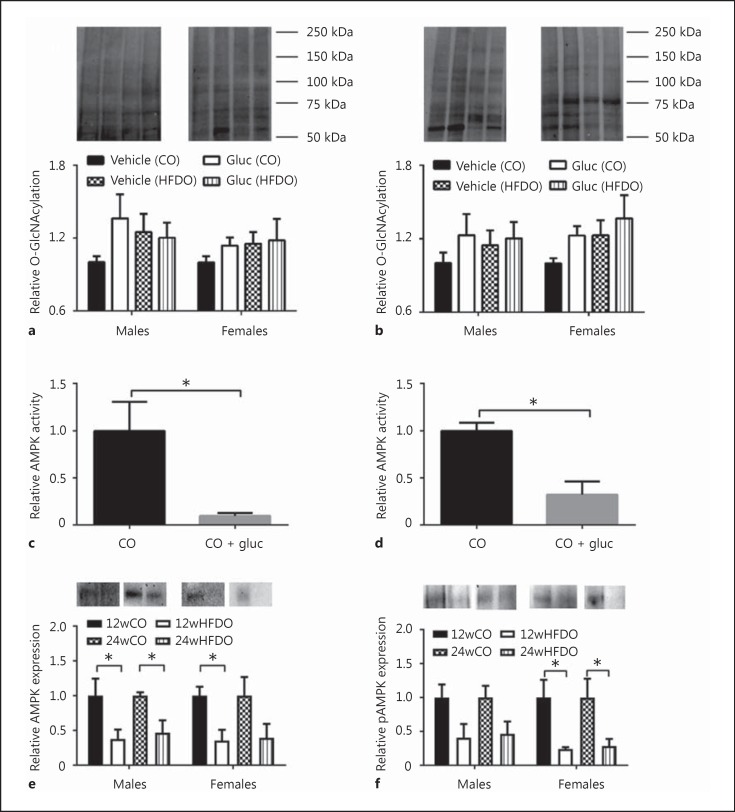
Glucosamine (gluc)-enhanced O-GlcNAcylation in PVAT. Pre-incubation with gluc increased total protein O-GlcNAcylation in both control and HFD PVAT from 12- (**a**) and 24-week-old (**b**) male and female offspring. Basal AMPK activity was reduced in control PVAT incubated with gluc from 24-week-old male (**c**) and female (**d**) offspring. AMPK (**e**) and phosphorylated AMPK (**f**) expressions were markedly reduced in male and female PVAT from 12- and 24-week-old HFDO. Representative immunoblots are shown in the upper part of each section; data are expressed as the mean ± SEM, *n* = 4; * *p* < 0.05, 1-way ANOVA/paired *t* test.

O-GlcNAcylation of protein was increased in the arteries from male 12wHFDO compared to controls (Fig. 5a), and there was also a trend towards an increase in female 12wHFDO arteries (Fig. 5b). Global protein O-GlcNAcylation appeared to be elevated in PVAT from both male and female HFDO at both ages compared to controls, although this was not statistically significant (Fig. 6a, b). There was also a trend towards increased O-GlcNAcylation in PVAT from controls incubated with glucosamine, but glucosamine did not increase it further in PVAT from HFD offspring (Fig. 6a, b).

Basal AMPK activity was reduced in PVAT from male 24wHFDO compared to controls (0.37 ± 0.04 vs. 1.00 ± 0.31, respectively; *p* < 0.05, *n* = 4, unpaired *t* test). There was also a trend towards reduced AMPK activity in PVAT from female 24wHFDO compared to controls (0.60 ± 0.15 vs. 1.00 ± 0.09, *p* < 0.07, *n* = 4, unpaired *t* test). Incubation of PVAT from male and female 24wCO with glucosamine also decreased AMPK activity (Fig. 6c, d). AMPK expression was decreased in male and female 12wHFDO as well as male 24wHFDO compared to controls; there was also a trend towards decreased AMPK expression in female 24wHFDO (Fig. 6e). Phosphorylated AMPK expression was reduced in male and female 24wHFDO compared to controls; there was also a trend towards decreased phosphorylated AMPK expression in male and female 12wHFDO compared to controls (Fig. 6f).

Phosphorylated eNOS expression was reduced in male 12wHFDO but not in females (online suppl. Fig. 5A, B). eNOS expression levels were similar in all of the groups apart from 24-week-old females, in which eNOS expression was reduced in HFDO compared to controls (online suppl. Fig. 5C, D).

## Discussion

Rat maternal obesity during pregnancy clearly has consequences for the offspring, not only with respect to their own body weight, but also by inducing epigenetic modifications which lead to detrimental cardiovascular effects [37]. In a recent study we found that maternal obesity led to PVAT dysfunction in male offspring [23]. In the present study we have extended those findings to demonstrate attenuation of the anticontractile effect of PVAT in both male and female offspring of rats fed a HFD. Moreover, we propose that, at least in the male offspring, PVAT dysfunction could be due to increased O-GlcNAcylation of eNOS within the PVAT, which reduces eNOS phosphorylation and consequently decreases NO bioavailability. Basal AMPK activity in PVAT from male and female HFDO was also reduced and this could also have contributed to the loss of the anticontractile effect. Nevertheless, activation of AMPK within HFDO PVAT partially restored its anticontractile properties.

Obesity is associated with the development of insulin resistance, the metabolic syndrome, and increased blood pressure [16], all of which are major risk factors for cardiovascular disease. The HFD provided to the rats prior to and during pregnancy resulted in a significantly increased body weight and arterial blood pressure compared to controls, effects typically observed in human obesity. There was also a marked trend towards increased plasma insulin levels in HFD dams, although this did not reach statistical significance.

In addition to the deleterious effects of obesity on women's health [38], maternal obesity also affects the next generation [39]. Offspring of obese mothers are at increased risk of developing obesity, glucose intolerance, and cardiovascular dysfunction [7, 9, 11]. This influence of the maternal environment on offspring health has led to the concept of “foetal programming of disease” [5, 6, 21]. Although the present study was not powered to assess body weight or arterial blood pressure as main outcomes, body weight was increased in the female 12- and 24wHFDO, and the male 24wHFDO compared to controls. Arterial blood pressure was also elevated from week 12 onwards in female HFDO but only from week 20 in male HFDO. These data show that the changes, which occurred as a result of foetal programming, are evident earlier in females than males, which is consistent with a previous study [7]. Adiposity was increased in both female and male HFDO at both ages. Plasma insulin levels were elevated in both male and female 24wHFDO and there was also a trend towards an increased serum insulin concentration in male and female 12wHFDO compared to controls, again consistent with previous findings [7]. Thus, in the present study, the maternal HFD had clearly predisposed the rat offspring to metabolic dysfunction, and this was apparent earlier in female offspring.

Vascular contractility is regulated by the endothelium which releases both vasodilator and vasoconstrictor agents. In the healthy state, an appropriate level of vascular tone is determined by the fine-tuning of their release [40], whereas obesity causes endothelial dysfunction which itself leads to further cardiometabolic disturbance [12, 13]. Endothelial dysfunction, which in the present study was evident in male 12- and 24wHFDO, has previously been linked to decreased NO bioavailability [14], which was also evident in this study. However, endothelial function was preserved in female offspring of the HFD dams, possibly due to the beneficial effect of female sex hormones [41] as eNOS phosphorylation was similar between HFDO and controls. Oestrogen reduces arterial contractility by increasing the endothelial release of NO and prostacyclin, and enhancing endothelium-dependent myocyte hyperpolarisation [42]. Therefore, in the present study, female offspring were sacrificed during the oestrous stage of their cycle to reduce variability due to hormonal influences.

PVAT is recognised as an endocrine organ that secretes a number of adipokines [43] that modulate vascular function [11]. PVAT exerts an anticontractile effect in healthy vessels through the release of PVAT-derived relaxant factors [22], but in obesity, impaired adipokine production, inflammation, and oxidative stress are proposed to be responsible for the reduced anticontractile effect of PVAT [21, 22]. Our group previously demonstrated that this anticontractile effect of PVAT is lost in male offspring of HFD dams, in part at least due to the decreased availability of NO from PVAT [23], although the underlying mechanism was not identified.

Increased protein O-GlcNAcylation has been linked to the vascular dysfunction associated with insulin resistance [24]. Increased plasma insulin, indicative of insulin resistance, was observed in the 24wHFDO and this was likely to have stimulated increased glucose uptake into adipocytes and vascular smooth muscle via an insulin-dependent glucose transporter (GLUT4), which is not present on the endothelium [44]. By increasing the end product of the hexosamine biosynthetic pathway, the elevated glucose concentration enhances protein O-GlcNAcylation. Since O-GlcNAcylation of eNOS reduces its activity [26], we hypothesised that the vascular and PVAT dysfunction in the offspring of obese mothers would be associated with increased protein O-GlcNAcylation.

Vascular contractions were reduced in PVAT-denuded arteries from male and female 12wHFDO compared to controls, an effect mimicked in control arteries by pre-incubation with glucosamine to increase O-GlcNAcylation. Since global protein O-GlcNAcylation was increased in mesenteric arteries from male 12wHFDO and there was a trend towards increased protein O-GlcNAcylation in female 12wHFDO arteries, it is possible that enhanced O-GlcNAcylation was responsible for the reduced vascular tone in HFDO. In contrast, other studies have reported augmented contractile responses due to increased O-GlcNAcylation. The reason for this discrepancy is unknown; however, in contrast to the resistance arteries used in the present study, conduit arteries were used in studies in which O-GlcNAcylation enhanced contractions [45, 46]. Surprisingly, the attenuated vascular contractions were only observed in younger HFDO and were not altered in arteries from 24wHFDO contracted with U46619, suggesting the development of a compensatory mechanism at a later age.

In order to determine the effect of O-GlcNAcylation on PVAT, the adipose tissue was removed from the artery and incubated separately with glucosamine (both with and without A769662), before being returned to the myograph bath containing the PVAT-denuded artery segment. This procedure allowed pharmacological modulation within PVAT without directly influencing the vascular tone of the endothelium-intact, PVAT-denuded artery segments. Under control conditions, returning the “exogenous” PVAT from control male and female offspring (of both ages) to the myograph baths containing their corresponding PVAT-denuded vessels, reduced contractions to U46619, indicating the release of a transferable relaxant factor. As expected, there was no effect of ePVAT from HFDO. Thus, this protocol clearly demonstrates a diminished anticontractile effect of PVAT due to maternal obesity preprogramming.

Pre-incubation of PVAT (from both male and female control offspring) with glucosamine (to stimulate O-GlcNAcylation) diminished its anticontractile properties. Moreover, glucosamine-pretreated PVAT increased vascular U46619-induced contractions in PVAT-denuded arteries from male offspring of both ages, suggesting it enhanced the release of a contractile factor, which is also observed in the procontractile male HFDO PVAT. Stimulation of the dysfunctional PVAT, from 12wHFDO and male 24wHFDO, with glucosamine had no effect on vascular contraction, but in female 24wHFDO it led to a greater increase in contractions compared controls. This is probably due to augmented O-GlcNAcylation in HFD PVAT compared to control PVAT and the fact that the PVAT dysfunction was not as prominent in female 24wHFDO. There was a trend towards increased total protein O-GlcNAcylation in both control PVAT incubated with glucosamine and HFD PVAT from male and female offspring at both ages, which further suggests that increased O-GlcNAcylation might be responsible for the loss of the anticontractile effect in the HFDO. Thus, we propose that by stimulating glucose influx into adipocytes, the elevated serum insulin levels increased protein O-GlcNAcylation in PVAT and that this resulted in its attenuated anticontractile effect. Oxidative stress has been previously linked with obesity-related PVAT dysfunction; it also stimulates the Hexosamine biosynthetic pathway and subsequently increases protein O-GlcNAcylation [47]. Therefore, the observed changes in this study could be due to elevated oxidative stress and thus protein O-GlcNAcylation.

O-GlcNAcylation also modulates the activity of various proteins [48] involved in the modulation of vascular contractility. O-GlcNAcylation of AMPK decreases its activity [49] and subsequently might reduce its vasodilator effects, which are attributed to eNOS phosphorylation [33]. In fact, glucosamine reduced basal AMPK activity within PVAT in older offspring; decreased AMPK activity was also observed in HFDO. In addition to this, O-GlcNAcylation of eNOS has been reported to attenuate NO bioavailability [26] and we have previously shown that the reduced anticontractile effects of PVAT in the male offspring of obese dams are possibly due to reduced NO bioavailability within PVAT [23]. Thus, we hypothesised that AMPK activation within PVAT might compensate for the loss of AMPK expression and that the consequent increase in eNOS phosphorylation could restore the anticontractile effect of PVAT in HFDO. Indeed, AMPK activation within HFD PVAT resulted in a partial restoration of its anticontractile effect on artery segments from both male and female HFDO at both ages. In PVAT from controls, simultaneous AMPK activation during the period of incubation with glucosamine (presumably by increasing NOS phosphorylation and thus reducing potential O-GlcNAcylation sites) prevented the loss of anticontractile effect in arteries from males but not females. By enhancing eNOS phosphorylation, AMPK activation would be expected to enhance NO release from both male and female PVAT. However, glucosamine only caused a reduction in the relative proportion of eNOS, which was phosphorylated in PVAT from male but not female offspring, suggesting that eNOS phosphorylation in females is maintained by an additional mechanism. AMPK expression was decreased in PVAT from both male and female HFDO, which further suggests that the loss of anticontractile effect of PVAT is due to reduced AMPK activity within HFD PVAT.

Collectively, these data indicate that maternal obesity leads to cardiometabolic effects in both male and female offspring. In particular, there was a marked reduction in AMPK expression and activity in the PVAT and the consequence of this was a reduction in the anticontractile effect of PVAT (which could be overcome by exposure to A769662, an AMPK activator). The reduced AMPK activity may have been a contributory factor in the development of the mild hypertension observed in the HFD offspring as PVAT has been recently shown to contribute to the control of blood pressure [50]. AMPK is known to increase eNOS phosphorylation and subsequently NO bioavailability. The loss of the anticontractile effect of HFDO PVAT seemed to be related to the reduced AMPK activity, but it did not correlate with a reduction in eNOS phosphorylation on Ser1177, suggesting a possible role for AMPK phosphorylation of other sites on eNOS or other NOS subtypes. The similarities between PVAT from control offspring that had been incubated with glucosamine and HFDO PVAT suggest that enhanced O-GlcNAcylation was likely to have played a role in the loss of the anticontractile effect of PVAT.

## Disclosure Statement

The authors have no conflicts of interest to declare.

## Supplementary Material

Supplementary data
