# Supplementary material for: The Role of O-GlcNAcylation in Perivascular Adipose Tissue Dysfunction of Offspring of High-Fat Diet-Fed Rats
Source: J Vasc Res. 2017 Apr 4;54(2):79–91. doi: 10.1159/000458422 (PMC5569708; doi:10.1159/000458422)
Supplement: Supplementary file 1 — Supplementary data [file jvr-0054-0079-s01.pdf]

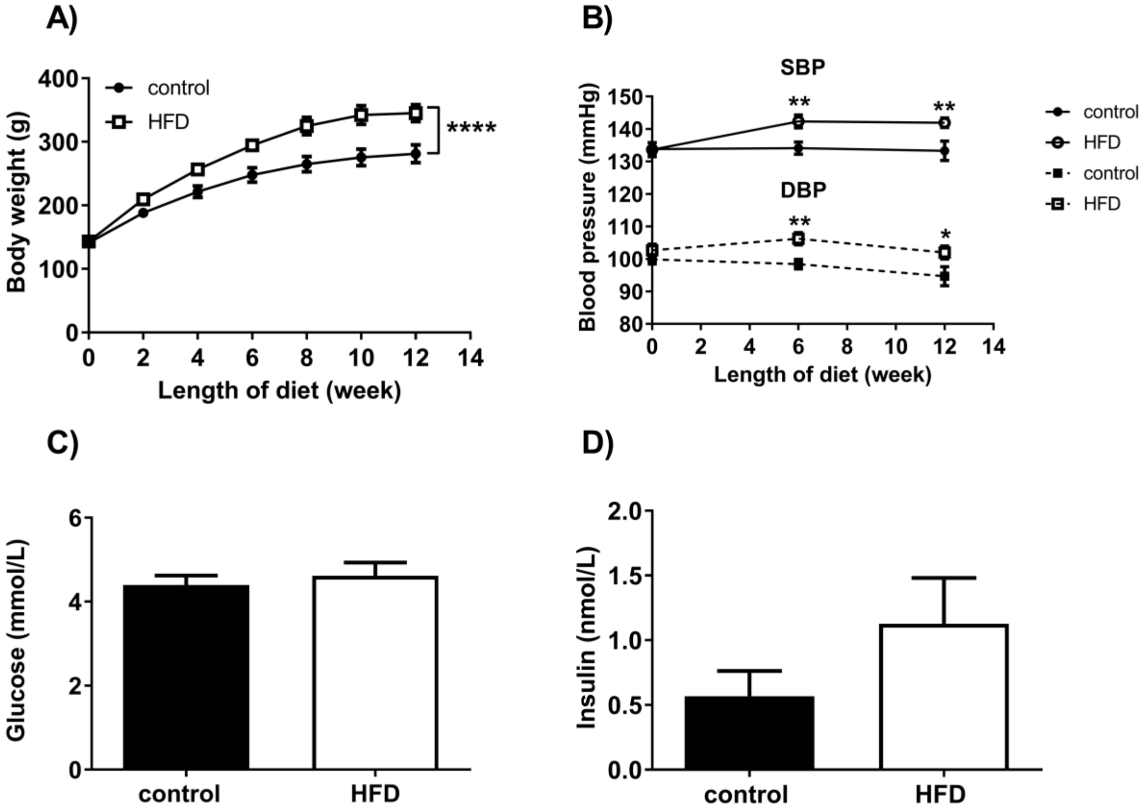

Supplementary figure 1. Maternal characteristics. Prior to pregnancy, body weight (A) and arterial blood pressure (B) were increased in HFD dams compare to controls. Blood glucose (C) was similar in control and HFD dams but there was a trend towards increased insulin (D) levels in HFD dams compared to controls. Data are expressed as mean±SEM, n=5, * p<0.05, ** p<0.01, **** p<0.0001, unpaired t-test.

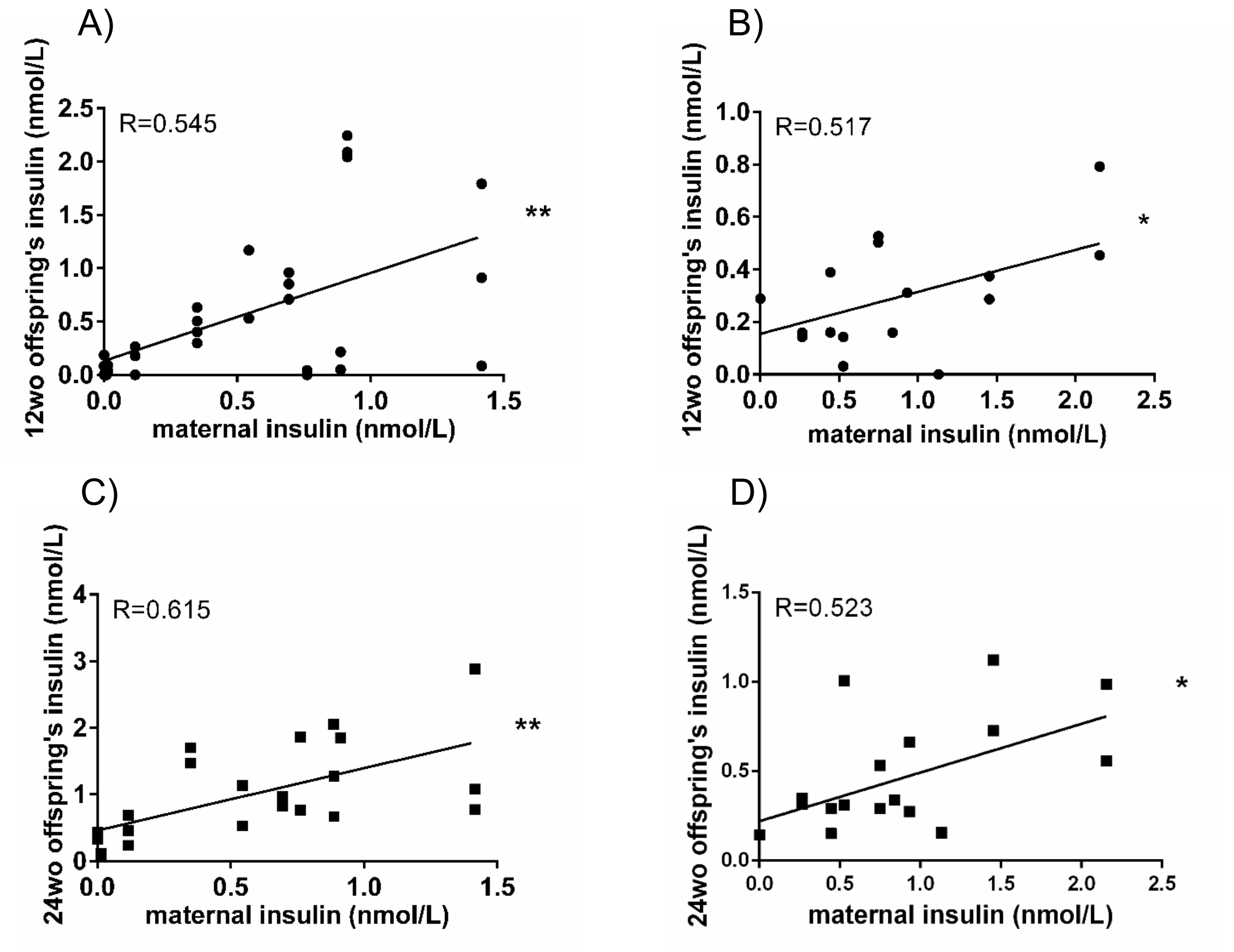

Supplementary figure 2. Maternal plasma insulin concentration positively correlated with 12 week old male (A) and female (B) offspring's insulin levels. Maternal plasma insulin levels also positively correlated with 24 week old male (C) and female (D) offspring's insulin concentration. N=14-20, * $p<0.05$, ** $p<0.01$, non-linear regression, R=Pearson correlation coefficient.

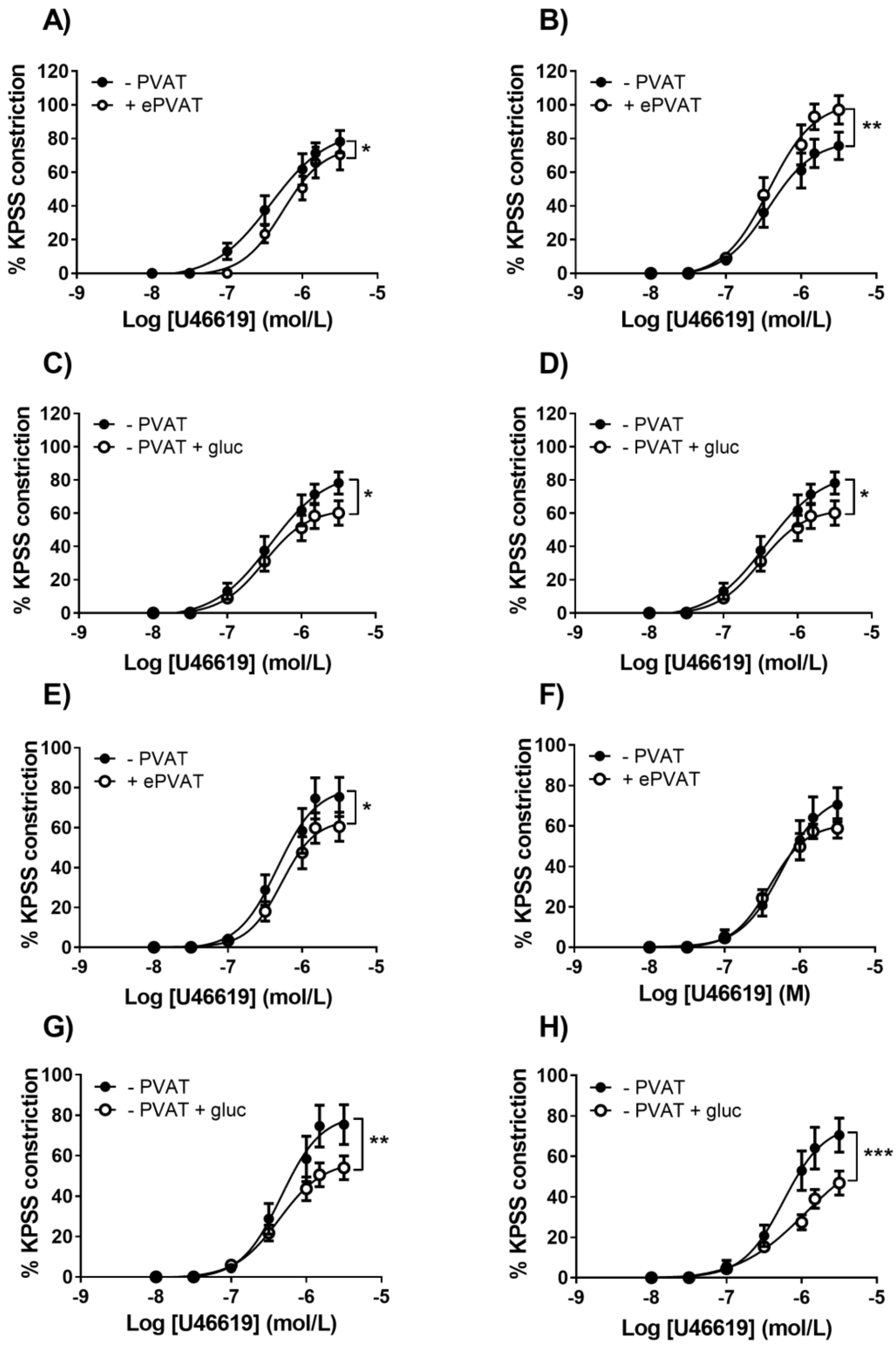

Supplementary figure 3. Loss of the anti-contractile effect of PVAT in 24 week old offspring of HFD dams. Exogenous PVAT (ePVAT) reduced vascular contractions to U46619 in arteries from 24 week old male (A) and female (E) offspring of control dams but not in vessels from male (B) and female (F) HFD offspring. Pre-incubation with glucosamine (10mM, gluc) reduced contractions in PVAT-denuded vessels from both male (C) and female (G) control as well as male (D) and female (H) HFD arteries. Data are expressed as mean±SEM, n=10, * $p<0.05$, ** $p<0.01$, *** $p<0.001$, two-way ANOVA. A769662 (A76). N=number of animals used.

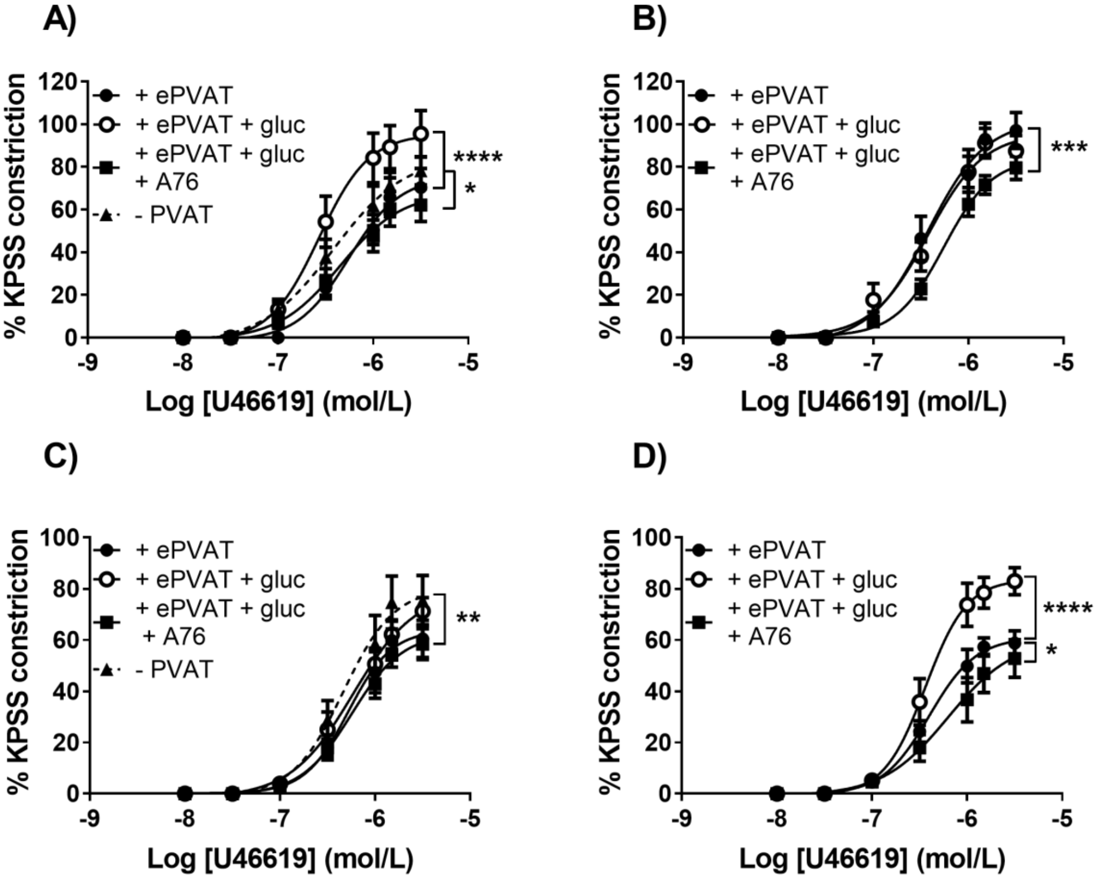

Supplementary figure 4. AMPK activation within PVAT partially restored the anti-contractile effect of PVAT in 24 week old offspring of HFD dams. After incubation of male exogenous PVAT (ePVAT) with 10mmol/L glucosamine, control (A) but not HFD ePVAT (B) increased vascular contractions in the corresponding PVAT-denuded artery segments. AMPK activation (with A769662) within glucosamine-treated control (A) and HFD (B) male ePVAT restored its anti-contractile effect. Preincubation of female control and HFD ePVAT with glucosamine had no effect on contractions to U466619 in the corresponding PVAT-denuded control vessels (C) but increased contractions in HFD (D) vessels. AMPK activation (with A769662) within glucosamine-treated HFD female ePVAT restored the anti-contractile properties of PVAT (D). Data are expressed as mean±SEM, n=10, * $p<0.05$, *** $p<0.001$, **** $p<0.0001$, two-way ANOVA. A769662 (A76). N=number of animals used.

	- PVAT		ePVAT		- PVAT + glucosamine		ePVAT + glucosamine		ePVAT + lucosamine + A76	
	Emax	pD2	Emax	pD2	Emax	pD2	Emax	pD2	Emax	pD2
12wCO (f)	73.18 ± 6.89	-6.27 ± 0.07	54.48 ± 8.22*	-6.19 ± 0.07	50.43 ± 5.51*	- 6.18 ± 0.07	63.36 ± 7.58^{+}	-6.21 ± 0.16	55.48 ± 6.85*	-6.23 ± 0.07
12wHFDO (f)	58.64 ± 9.71	-6.26 ± 0.11	81.11 ± 18.70	-6.10 ± 0.07	49.78 ± 6.12	-6.21 ± 0.16	79.47 ± 10.61	-6.19 ± 0.09	58.71 ± 9.11	-6.10 ± 0.05
12wCO (m)	102 ± 18.03	-6.39 ± 0.12	63.12 ± 5.73**	-6.41 ± 0.09	71.72 ± 9.89*	-6.36 ±0.08	95.35 ± 10.85$^{\#\#}$	-6.20 ± 0.09	65.98 ± 14.00$^{\#}$	-6.20 ± 0.06
12wHFDO (m)	72.86 ± 5.97	-6.23 ± 0.06	92.77 ± 10.57*	-6.25 ± 0.08	62.65 ± 5.99	-6.27 ± 0.07	78.82 ± 6.14	-6.14 ± 0.08	71.87 ± 8.27$^{\#}$	-6.26 ± 0.06

Supplementary table 1. Emax and pD2 values. pD2 values expressed as mean±SEM, Emax values expressed as mean±SEM (% KPSS), n=10, -PVAT * p<0.05, ** p<0.01, + p=0.0566, + PVAT # p<0.05, ## p<0.01, paired t-test.

	- PVAT		ePVAT		- PVAT + glucosamine		ePVAT + glucosamine		ePVAT + glucosamine + A76	
	Emax	pD2	Emax	pD2	Emax	pD2	Emax	pD2	Emax	pD2
24wCO (f)	82.40 ± 10.45	-6.30 ± 0.07	64.44 ± 6.86^{+}	-6.28 ± 0.09	82.40 ± 10.45	-6.33 ± 0.09	81.49 ± 9.34$^{\&}$	-6.25 ± 0.10	62.06 ± 7.07*	-6.25 ± 0.09
24wHFDO (f)	77.93 ± 11.21	-6.15 ± 0.16	65.25 ± 4.35	-6.31 ± 0.07	77.93 ± 11.21	-6.22 ± 0.07	85.92 ± 5.88$^{\#\#}$	-6.38 ± 0.08	55.89 ± 7.91*	-5.92 ± 0.14$^{\#}$
24wCO (m)	87.17 ± 7.80	-6.37 ± 0.13	73.80 ± 9.64	-6.29 ± 0.08	87.17 ± 7.80	-6.41 ± 0.08	101.30 ±9.48$^{\#}$	-6.46 ± 0.11	66.08 ± 7.72*	-6.32 ± 0.09
24wHFDO (m)	83.31 ± 6.40	-6.29 ± 0.12	103.20 ± 7.01*	-6.40 ± 0.10	83.31 ± 6.40	-6.38 ± 0.08	97.42 ± 9.06	-6.44 ± 0.08	81.69 ± 5.16$^{\#}$	-6.28 ± 0.07

Supplementary table 2. Emax and pD2 values. The pD2 value for contraction to U46619 was reduced by the presence of exogenous PVAT (ePVAT) pre-incubated with glucosamine and the AMPK activator A769662 (A76) compared to ePVAT from 24 week old female (f), but not male (m) offspring of HFD dams (24wHFDO). Data are expressed as mean±SEM, Emax values expressed as mean±SEM (% KPSS), n=10, -PVAT * p<0.05, + p<0.07, ePVAT # p<0.05, ## p<0.01, & p=0.0651, paired t-test.

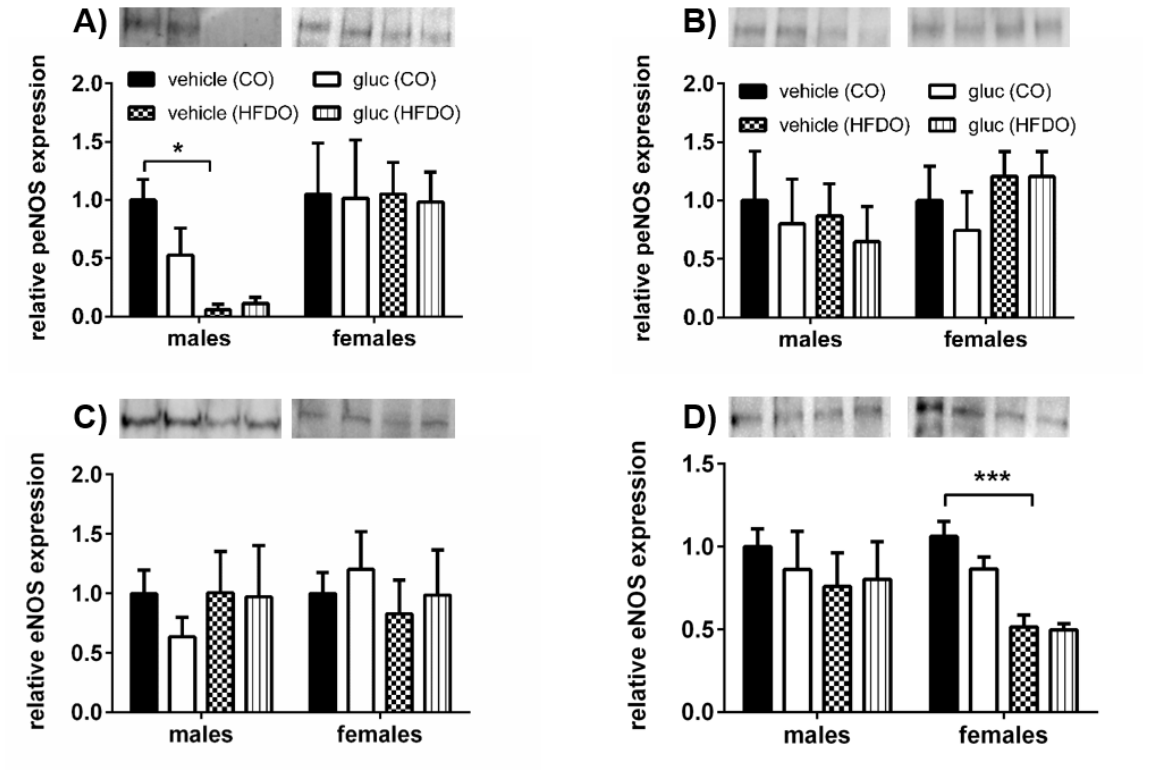

Supplementary figure 5. Phosphorylated eNOS expression in reduced in PVAT from HFDO. Phosphorylated eNOS expression was reduced in PVAT from 12 (A) week old male offspring but not in females or 24 week old male offspring (B). Total eNOS expression was similar in control and HFD PVAT (C) from 12wo female and male offspring but in was decreased in 24wo (D) female HFDO. Representative immunoblots are shown in the upper part of each section; data are expressed as mean±SEM, n=4, * $p<0.05$, one-way ANOVA. N=number of animals used.
